# Impact of Leptospermone, a Natural β-Triketone Herbicide, on the Fungal Composition and Diversity of Two Arable Soils

**DOI:** 10.3389/fmicb.2019.01024

**Published:** 2019-05-10

**Authors:** Clarisse Mallet, Sana Romdhane, Camille Loiseau, Jérémie Béguet, Fabrice Martin-Laurent, Christophe Calvayrac, Lise Barthelmebs

**Affiliations:** ^1^Laboratoire Microorganismes : Génome et Environnement, CNRS, Université Clermont Auvergne, Clermont-Ferrand, France; ^2^AgroSup Dijon, INRA UMR1347 Agroécologie, Université de Bourgogne, Université Bourgogne Franche-Comté, Dijon, France; ^3^Biocapteurs-Analyses-Environnement, Université de Perpignan Via Domitia, Perpignan, France; ^4^Laboratoire de Biodiversité et Biotechnologies Microbiennes, USR 3579 Sorbonne Universités (UPMC) Paris 6 et CNRS Observatoire Océanologique, Banyuls-sur-Mer, France

**Keywords:** fungal community, microbial ecotoxicology, bioherbicide, leptospermone, soil

## Abstract

Impact of leptospermone, a β-triketone bioherbicide, was investigated on the fungal community which supports important soil ecological functions such as decomposition of organic matter and nutrients recycling. This study was done in a microcosm experiment using two French soils, Perpignan (P) and Saint-Jean-de-Fos (SJF), differing in their physicochemical properties and history treatment with synthetic β-triketones. Soil microcosms were treated with leptospermone at recommended dose and incubated under controlled conditions for 45 days. Untreated microcosms were used as control. Illumina MiSeq sequencing of the internal transcribed spacer region of the fungal rRNA revealed significant changes in fungal community structure and diversity in both soils. Xylariales, Hypocreales, Pleosporales and Capnodiales (Ascomycota phyla) fungi and those belonging to Sebacinales, Cantharellales, Agaricales, Polyporales, Filobasidiales and Tremellales orders (Basidiomycota phyla) were well represented in treated soil microcosms compared to control. Nevertheless, while for the treated SJF a complete recovery of the fungal community was observed at the end of the experiment, this was not the case for the P treated soil, although no more bioherbicide remained. Indeed, the relative abundance of most of the saprophytic fungi were lower in treated soil compared to control microcosms whereas fungi from parasitic fungi included in Spizellomycetales and Pezizales orders increased. To the best of our knowledge, this is the only study assessing the effect of the bioherbicide leptospermone on the composition and diversity of the fungal community in soil. This study showed that leptospermone has an impact on α- and β-diversity of the fungal community. It underlines the possible interest of microbial endpoints for environmental risk assessment of biopesticide.

## Introduction

The use of biopesticides, defined as natural active compounds, is viewed as a safe alternative to agrochemicals for crop protection as they are considered to be less harmful and environmental-friendly compared to synthetic pesticides (Dayan et al., 2007, 2011; Seiber et al., 2014). Nowadays, only thirteen bioherbicides are commercially available (Cordeau et al., 2016). Among compound of interest, leptospermone [2,2,4,4-tetramethyl-6-(3-methyl-1-oxobutyl)-1,3,5-cyclohexanetrione], an allelopathic compound isolated from bottlebrush plant (*Callistemon citrinus*) was used as pre-and post-emergence herbicide to control broad leaf and grass weeds at a rate of 3 L ha^−1^ (Dayan et al., 2011). Belonging to β-triketone family, leptospermone inhibits the 4-hydroxyphenylpyruvate dioxygenase (HPPD), a key enzyme in plant carotenoid biosynthesis, (Rocaboy-Faquet et al., 2014) leading to photosynthetic chlorophyll destruction of plant (Lee et al., 2008). However, HPPDs are not specific for plant and are found in prokaryotes and fungi, including those living in soil (Keon and Hargreaves, 1998). Soil microorganisms which are known to accomplish numerous functions supporting soil ecosystemic services are key drivers to be protected (EFSA, 2017). As recently underlined by Amichot et al. (2018) the side effect of biopesticides has to be addressed, particularly assessing their environmental fate and impact on soil microorganisms (Gopal et al., 2007; Gupta et al., 2013; Singh et al., 2015a,b; Romdhane et al., 2016), and to the best of our knowledge no studies have been performed to estimate the effect of biopesticides on fungal community (Thomson et al., 2015). Fungi are known to play fundamental ecological roles in natural and managed agricultural soils (Al-Sadi et al., 2015; Tardy et al., 2015). As it was previously observed, application of pesticides, even at the recommended field dose, can induce a reduction in the overall fungal community, that could impacted the ecosystem services in which fungi are involved (Sebiomo et al., 2011; Schlatter et al., 2018). On the other hand, pesticides can be used as carbon source by some fungi known to possess biodegradation abilities (Ikehata et al., 2004; Tortella et al., 2005; Coppola et al., 2011; Panelli et al., 2017; Góngora-Echeverría et al., 2018).

Within this context, this study assessed for the first time, the impact of the bioherbicide leptospermone on the structure and diversity of fungal community inhabiting two soil types. We tested the effect of leptospermone on the diversity and composition of the fungal communities during the experimental time and in a same manner in the two different soils. This goal was reached by high throughput deep sequencing [Illumina MiSeq-based amplicon sequencing of ribosomal internal transcribed spacer 2 (ITS2) region]. The richness of each sample (α-diversity) and the diversity in composition among samples (β-diversity) were assessed over time.

**Table 1:** Physico-chemical characteristics of the two soils (P and SJF) used for the experiment.

Soil characteristics	P Soil	SJF Soil
Clay	13.9%	25.8%
Silt	60.5%	27.3%
Sand	25.6%	46.9%
Soil humidity,	20%	15%
Organic matter	1.7%	1.5%
Organic carbon	0.98%	0.9%
Cation exchange capacity (CEC)	15.5 meq 100 g^−1^	10.4 meq 100 g^−1^
Ca^2+^/CEC	214%	98%
pH in water	8.1.	7.62
History	Experimental field site having a β-triketone history treatment	Neither cultivated nor treated with pesticides for the last 5 years

## Materials and Methods

### Soil Characteristics and Microcosm Experiment

Soils and microcosm set-up was previously described in Romdhane et al. (2016). The two soils present different textures with 2-fold more clay and sand proportion in Saint-Jean-de-Fos (SJF) than in Perpignan (P) and with 2-fold less silt in SJF than in P (Table 1). Both soils are poor in organic matter and show a moderate cation exchange capacity.

Pure leptospermone was obtained as described by Owens et al. (2013). Soil microcosms were treated at recommended field dose (D1, 5 μg g^−1^) or not (D0, control). For each soil, three randomly chosen microcosms were sacrificed by sampling date and treatment (*n* = 12 per sampling date). Soil samples were then subdivided in different aliquots for dissipation studies and molecular analysis.

### Fate of Leptospermone in the Soil Microcosms

Kinetics of dissipation and adsorption observed in P and SJF soils have already been reported in Romdhane et al. (2016). Briefly, leptospermone dissipation was measured during the time course of the incubation. Soil extracts were prepared from 10 g of each soil, and analyzed by HPLC/UV (Romdhane et al., 2016). Calibration curves prepared with spiked blank soil samples, were used to determine the leptospermone in soil extracts, with a quantification limit of 0.2 mg L^−1^.

Adaptation of the batch equilibrium method was applied to determine adsorption isotherms of leptospermone to both soils, using soil samples spiked with a range of leptospermone solutions (1 to 40 mg L^−1^) (Wilson and Chester, 1992; OECD, 2000; Romdhane et al., 2016). After 3 h of agitation with 1 g of soil sample, remaining leptospermone in solution was measured by HPLC/UV.

### Composition and Diversity of the Fungal Community

The composition and diversity of the fungal community were analyzed from DNA extracts [ISO 11063 derived from Martin-Laurent et al. (2001)] for P and SJF soils, treated (D1) or not (D0) with leptospermone at days 0, 4 and 45.

The amount of DNA was quantified (NanoDrop Technologies, DE, United States) and standardized to 10 ng μL^−1^. Aliquots were used as template to amplify Internal Transcribed Spacer 2 (ITS2) region of the ribosomal RNA gene cluster (Ihrmark et al., 2012; Tedersoo et al., 2014; Oliver et al., 2015). Amplicons were amplified with fITS7 (5′-GTGARTCATCGAATCTTTG-3′, Ihrmark et al., 2012) and rITS4 (5′-TCCTCCGCTTATTGATATGC-3′, White et al., 1990) supplemented with overhang adapter at the 5′ end of each primer. A PCR was performed in 25 μL reaction volume with the following concentrations: DNA 30 ng, 300 μM dNTPs, 0.4 μM of both primers (fITS7, rITS4), 1× Kapa Hifi HotStart ReadyMix containing 2.5 mM MgCl_2_ and 0.5 unit of Kapa Hifi HotStart DNA polymerase (KapaBiosystems, United States). PCR cycling parameters were: 95°C for 3 min, plus 30 cycles at 98°C for 20 s, 57°C for 30 s, and 72°C for 30 s, and followed by an extension at 72°C for 5 min. Relative quantity and yield were checked on 1% agarose gel and quantified using the Quant-iT^TM^ PicoGreen^TM^ dsDNA Assay Kit (Thermo Fisher Scientific, United States). The resulting PCR products were sent to the GeT-PLaGe platform (INRA, France) for multiplexing, purification and sequencing on the Illumina MiSeq platform.

### Sequence Analysis

Reads were passed through a quality control filter that removes sequences with more than one expected errors for all bases in the read and sequences under a minimum length threshold (100 bp). Then, a dereplication step was performed: set of unique sequence were find, sequences were compared letter-by-letter and must be identical over the full length of both sequences. In this step, singletons were discarded. UNOISE algorithm (Edgar and Flyvbjerg, 2015) was performed to denoise amplicon. Reads with sequencing error were identified and corrected and chimeras were removed. High-quality reads were then clustered into operational taxonomic units (OTUs) at 97% similarity using UPARSE (Edgar, 2013). The taxonomic assignment to species level was carried out using SINTAX algorithm (Edgar, 2016) against the Utax 22.08.2016 database (Supplementary Table S1).

α- and β-diversity indexes were assessed at the taxonomic rank of OTUs (MOTHUR, Schloss et al., 2009). Sample richness estimated by observed richness, Chao 1 index, the Shannon index and Simpson’s index were used to compare the soil fungal community α-diversity. To visually identify patterns of community structure among the samples (β-diversity), a Principal Coordinates Analysis (PCoA) analysis based on ThetayC dissimilarity matrix, was performed with MOTHUR (Schloss et al., 2009).

### Statistical Analysis

Differences between leptospermone treatments at days 0, 4 and 45 were assessed using a Kruskal–Wallis non parametric test (*P* < 0.05) using “nparcomp” package of R software (Konietschke et al., 2015). Sparse Partial Least Squares Discriminant Analysis (sPLS-DA) was performed to select discriminant OTUs between different treatments using the function “splsda” from R package mixOmics (Lê Cao et al., 2017).

**Table 2:** Richness and diversity indices of the fungal community calculated for P and SJF soils microcosms exposed to leptospermone applied at the agronomical dose (D1) or not (D0) at 0, 4 and 45 days (d0, d4, d45) (mean values ± standard deviation, *n* = 3).

Samples		Index			
		Number of OTUs (Observed richness)	Chao l	Shannon-Wiener (H’)	Simpson (D)
	P-D0-d0	471.3 ± 22.5a	559.5 ± 63.0a	4.38 ± 0.36a	0.96 ± 0.01a
	SJF-D0-d0	581.0 ± 86.6b	754.6 ± 120.0b	4.68 ± 0.04a	0.97 ± 0.00a
	P-D0-d45	378.0 ± 67.0a	501.4 ± 73.1a	4.65 ± 0.34a	0.98 ± 0.01a
	SJF-D0-d45	528.3 ± 41.4b	671.0 ± 69.8a	4.45 ± 0.08a	0.97 ± 0.00a
Perpignan	P-D0-d4	529.0 ± 5.5a	629.1 ± 31.3a	4.85 ± 0.10a	0.98 ± 0.00a
	P-Dl-d4	323.5 ± 90.5b	488.4 ± 135.8a	3.74 ± 0.40b	0.95 ± 0.01b
	P-D0-d45	378.0 ± 67.0a	501.4 ± 73.1a	4.65 ± 0.34a	0.98 ± 0.01a
	P-Dl-d45	493.7 ± 46.6a	679.3 ± 94.8a	3.85 ± 1.5a	0.81 ± 0.27a
Saint Jean de Fos	SJF-D0-d4	582.3 ± 20.9a	746.2 ± 80.3a	4.61 ± 0.18a	0.97 ± 0.01a
	SJF-Dl-d4	462.5 ± 55.7a	711.9 ± 81.4a	4.57 ± 0.10a	0.98 ± 0.00a
	SJF-D0-d45	528.3 ± 41.4a	671.0 ± 69.8a	4.45 ± 0.08a	0.97 ± 0.00a
	SJF-Dl-d45	411.0 ± 105.8a	517.0 ± 137.5a	4.34 ± 0.07a	0.97 ± 0.00a

*Different letters indicate significant differences between the initial or final fungal diversity of the two soils, and between leptospermone treatments (D1) compared to control (D0) at days 4 and 45.*

## Results

As previously observed by Romdhane et al. (2016), leptospermone applied at the agronomical dose in P and SJF soils was moderately adsorbed and in a similar manner for both soils with K_oc_ values of 144 and 137 mL g^−1^, respectively. The dissipation was significantly faster in P than in SJF soil (DT_50_ = 4 and 9 days, respectively) but for both soils, leptospermone was entirely dissipated at the end of the incubation.

A total of 1630991 reads (min: 14933, max: 57410) were obtained through NGS sequencing (MiSeq Illumina sequencing). After quality control, 553800 sequences remained for analyses. The number of sequences per samples were of 13845 for a total of 2278 OTUs which were obtained at 98% similarity threshold.

**FIGURE 1:**
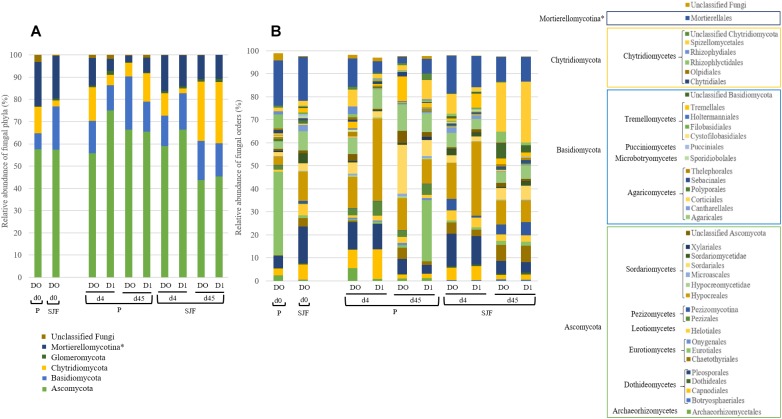
Relative abundance (expressed as % of the total number of OTUs) of fungal **(A)** phyla and **(B)** orders (level of class membership was also reported) in P and SJF soils microcosms exposed to leptospermone applied at the agronomical dose (D1) or not (D0) for 0, 4 and 45 days (d0, d4, d45). ^∗^represented subphylum level.

**FIGURE 2:**
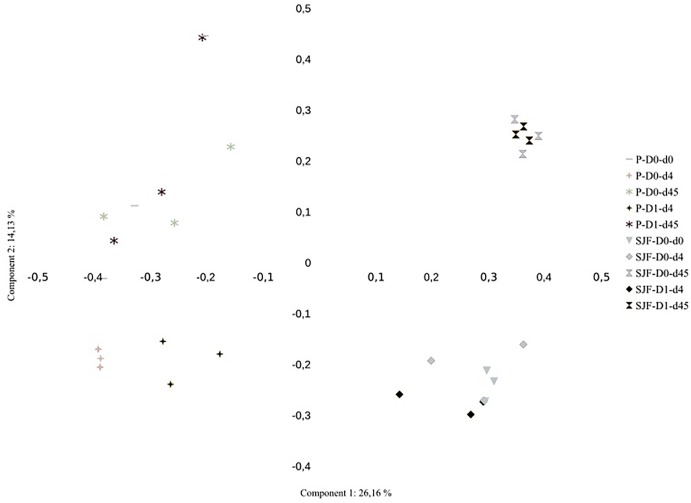
Principal Coordinate Analysis (PCoA) of the effect of leptospermone applied at the agronomical dose (D1) as compared to the control (D0) on the structure of the fungal community composition of P and SJF soils at 0, 4 and 45 days (d0, d4, d45). Percent of variance explained by the first two axis of the PCoA is given.

**FIGURE 3:**
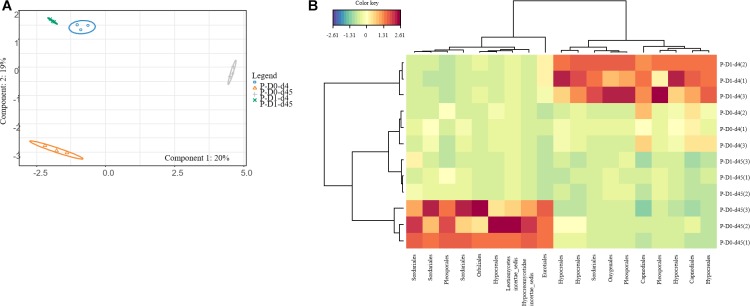
sPLSDA analysis **(A)** of the effect of leptospermone applied at the agronomical dose (D1) on the fungal community composition in P soil at 4 and 45 days (d4, d45) as compared to control (D0) (95% confidence level ellipse plots). Heatmap plot **(B)** representing the relative abundances of OTUs picked up using sPLSDA in P soil microcosms (numbers in brackets denote replicate).

**FIGURE 4:**
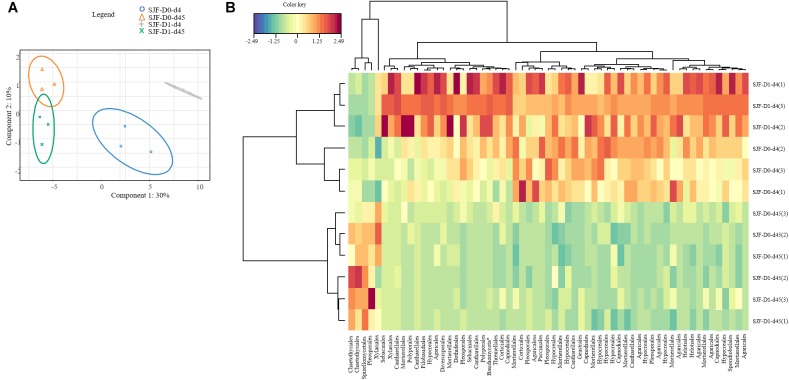
sPLSDA analysis **(A)** of the effect of leptospermone applied at the agronomical dose (D1) on the fungal community composition in SJF soil at 4 and 45 days (d4, d45) as compared to control (D0) (95% confidence level ellipse plots). Heatmap plot **(B)** representing the relative abundances of OTUs picked up using sPLSDA in SJF soil microcosms (numbers in brackets denote replicate).

Ascomycota was the most dominant phylum (P: 58%, SJF: 57%), with mainly Sordariomycetes class (P: 26%, SJF: 38%) represented by Hypocreales (P: 4%, SJF: 13%) and Sordariales orders (P: 3%, SJF: 3%), Dothideomycetes (P: 35%, SJF: 41%) class with Capnodiales (P: 3%, SJF: 7%) and Pleosporales (P: 5%, SJF: 16%) orders and Eurotiomycetes class (P: 14%, SJF: 9%) represented mainly by order of Eurotiales (P: 36%, SJF: 1%) (Figure 1). Basidiomycota phylum (P: 7%, SJF: 19%) was represented by Agarocomycetes class (P: 78%, SJF: 77%) with Agaricales order (P: 3%, SJF: 9%) and Tremellomycetes class (P: 20%, SJF: 14%) with Tremellales order (P: 1%, SJF: 2%). Mortierellomycotina (P: 20%, SJF: 19%) subphylum was only represented by Mortierellales order. Chrytridiomycota (P: 12%, SJF: 3%) phylum was represented by one class Chytridiomycetes and mainly two orders (Rhizophlyctidales, P: 6% for, SJF: 0%, and Spizellomycetales, 2% for both soils) (Figure 1). The composition of the fungal community of P soil was different from that of SJF (Figure 1, 2). At both d0 and d45 observed richness was significantly lower in P than in SJF soil while Chao1 showed similar trend but only at D0 (Table 2). After 4 days of exposure, Shannon and Simpson indices of fungal communities recorded in P soil was lower than in those of the control (*p* = 0.049). Accordingly, at the same time, the composition of the fungal community observed in P soil after 4 days of treatment was clearly discriminated from the control (Figure 3A). In order to visualize the relative abundance of selected OTUs in different treatments, a clustered heatmap was generated for both soils. OTUs mainly affiliated to Hypocreales (*Fusarim* sp.) and Capnodiales (*Cladosporium* sp.) were significantly higher in leptospermone treated soil (Figure 3B). The relative abundance of Sordariales, Spizellomycetales and Mortierellales fungi decreased in the treated P soil (Figure 1). Forty five days after the treatment, fungal composition was still significantly different from the control mainly represented by Sordariales, Pleosporales, Hypocreales, leotiomycetes and Hypocreomycetidae fungi (Figure 3B) while the relative abundance of Eurotiales, Pezizales, Spiezellomycetales, Chytridiomycetales and Mortierellales fungi were higher in treated P soils (Figure 1). In the SJF soil, leptospermone did not induced significant changes in the α-diversity on the fungal community (Figure 2 and Table 2), contrary to what was observed for P soil. Nonetheless, significant changes in the β-diversity of the fungal community were recorded in SJF soil exposed microcosms as compared to control (Figure 4A). While Ascomycota OTUs dominated the composition of the fungal community of P soil, Basidiomycota and Zygomycota OTUs were dominant in SFJ soil (Figure 3B, 4B). In Figure 4B, one could observe that the relative abundance of OTUs, responsive to leptospermone exposure, affiliated to Xylariales and Hypocreales orders (Sordariomytes class), Dothideales and Pleosporales orders (Dothideomycetes class), Sebacinales, Cantharellales, Polyporales and Cortiales orders (Agaricomycetes class), Filobasisiales and Tremellales orders (Tremellomycetes class) and Mortierellales order (Zygomycota phylum) were significantly higher than in the control microcosms. At the end of the incubation (45 days), the fungal community of SJF treated soil was not significantly different from that of control (Figure 4A) and OTUs were mainly affiliated to Chaetothyriales and Spizellomycetales orders (respectively Eurotiomycetes and Chytridiomycetes classes) (Figure 4B).

## Discussion

To date, although EFSA recommended to implement microbial endpoints, such as arbuscular mycorrhiza fungi, in pesticide environmental risk assessment (EFSA, 2010), there are still a limited number of studies evaluating side-effect of synthetic pesticides on soil fungal community (Borowik et al., 2017; Morrison-Whittle et al., 2017; Rivera-Becerril et al., 2017; Góngora-Echeverría et al., 2018) and almost none concerning biopesticides (Gopal et al., 2007).

To the best of our knowledge, this is the first study assessing the effect of the leptospermone on the composition and diversity of the fungal community in two different arable soils. As expected, the composition of fungal community of P and SJF soils were differing. Indeed, whether it is through α-diversity index or through fungal community composition, P soil presented the lower species richness and diversity. Its composition was mainly dominated by Eurotiales and Mortierellales orders while in SJF soil, Pleosporales, Hypocreales and Mortierellales orders predominated. These differences could be partially due to different physicochemical characteristics of the two soils and in particular clay content, which is well known to influence the aggregate size, moisture content and pH (Vargas-Gastélum et al., 2015), key abiotic parameters of ecological niches. Indeed, higher fungal alpha diversity in SJF soil than in P soil and the presence of Pleosporales et Hypocreales fungi could be due to the fine-size particles in this soil clay fraction (Wang et al., 2016). Moreover, these first results could also be explained by the history treatment of the two soils. Indeed, SJF soil was neither cultivated nor treated with pesticides for the last 5 years contrary to P soil which was cropped with corn and treated with synthetic β-triketone herbicides (Romdhane et al., 2016), which could explained the higher diversity obtained in SJF soil. However, one could observe common features between the two soils such as the predominance of *Mortierella*. These highly opportunistic genera are able to rapidly grow on simple organic matter (Tardy et al., 2015) which could reflect easily decomposable C-substrates in the two assessed soils. A low abundance of Glomeromycota (<1% in both soils) was observed in both soils. This result has already been observed in other studies (Xu et al., 2012; Panelli et al., 2017) and may be due to the fact that this phylum, gathered genera which are obligate arbuscular mycorrhiza fungi forming symbiosis with roots of most of vascular plants, could be poorly covered by primers (Hartmann et al., 2015).

Dissipation of the bioherbicide was depending on the soil (P-DT50 < SJF-DT50): longer persistence of leptospermone was observed in SJF soil compared to P soil (Romdhane et al., 2016). However, after 4 days of exposure to the bioherbicide, significant changes in the structure of the fungal community were observed in both soils compared to the control. The increase of the relative abundance of Xylariales, Hypocreales (Sordariomycetes class), Pleosporales and Capnodiales (Dothideomycetes class) fungi might be linked to an increase of organic matter mineralization by fungal activity (Lienhard et al., 2014) and to their possible involvement in the biopesticide degradation, as previously shown for synthetic pesticides (Bell and Wheeler, 1986; Coppola et al., 2011). These orders of fungi grow quickly and become dominant because they are well adapted to metabolize low molecular weight organic carbon sources (Hannula et al., 2012) such as the compounds released by dead sensitive microorganisms like Spiezellomycetales, Pezizomycotina and Mortierellales fungi (respectively Chytridiomycetes, Pezizomycetes classes and Mucoromycotina subphylum). Moreover these fungi with melanin pigments in their hyphae have been associated with mitigating environmental stresses (including high temperatures, high UV radiation and extended drought) (Bell and Wheeler, 1986). Genera like *Fusarium* (Hypocreales order), well represented in treated soil microcosms, are among those that most significantly respond to the changing of agricultural managements (Hartmann et al., 2015) and could be much tolerant to xenobiotic (Bourgeois et al., 2015). Bioherbicide transformation might also explain the increase of the relative abundances of fungal OTUs related to Sebacinales, Cantharellales, Agaricales and Polyporales orders and Filobasidiales and Tremellales ones (respectively Agaricomycetes and Tremellomycetes classes) in SJF soil, where leptospermone was still at rather high concentration after 4 days of exposure as compared to P soil (Romdhane et al., 2016). Indeed, several genera from these orders (i.e., *Trametes* sp., *Phanerochaete* sp., *Cyathus* sp., *Phlebia* sp.*, Cryptococcus* sp.) are known to have relatively broad amplitude of ecological tolerance and to transform lignin and/or various recalcitrant organic pollutants including numerous pesticides (Singh et al., 1999; Singh and Kuhad, 1999; Ikehata et al., 2004; Tortella et al., 2005; Xiao et al., 2011; Johnson and Echavarri-Erasun, 2011; de Garcia et al., 2012). Moreover, these fungi, as *Mortierella* genus (Mortierellales order) can grow in contaminated soils with pesticides (Kataoka et al., 2010; Salar, 2012), also because they can use fresh organic matter released from dead microorganisms sensitive to pesticides (Tardy et al., 2015).

Interestingly, after 45 days of incubation, the fungal community of treated SJF soil was able to rapidly recover, probably because of the entire dissipation of leptospermone (Romdhane et al., 2016). Surprisingly, the β-diversity of fungal community did not fully recover in P soil, although leptospermone was dissipated more rapidly than in SJF soil. At the end of the incubation in P soil, one could observe that the relative abundance of most of the saprophytic fungi and particularly those belonging to Sordariales, Hypocreales Pleosporales, Helotiales, Agaricales and Tremellales orders were lower in treated soil compared to control microcosms whereas fungi from Eurotiales order and parasitic fungi included in Spizellomycetales and Pezizales orders increased. In this condition, the dissipation of the leptospermone might have an antagonist effect by stopping the pressure on parasitic fungi. This persistent changes in the fungal community might result from a system drift due to changes in the quantity and quality of organic matter in P soils induced by leptospermone exposure (Klaubauf et al., 2010; Ma et al., 2013; Wang et al., 2016).

To conclude leptospermone applied at the agronomical dose caused significant changes in the β-diversity of the fungal community. Nevertheless, while the fungal community completely recovered in the SJF soil as already observed for bacterial community (Romdhane et al., 2016), the recovery was not observed in the P treated soil, although no more bioherbicide remained. Contrary to the bacterial community (Romdhane et al., 2016), the resilience of the fungal community is not obtained for both soils even after the entire dissipation of leptospermone. Taking together, our results showed that characterization of effect of herbicides and/or bioherbicides on microbial community and ecosystem functions, have to integrate both bacterial and fungal communities. Indeed, treatments have shown to induce different responses and led to promotion or suppression of beneficial or pathogenic fungal taxa (Hartmann et al., 2015). However, only two soil types and one concentration of application were tested thereby burden with doubt any generalization. Further study is needed to distinguish between direct and indirect (e.g., drift of the system) effects of leptospermone. This study showed that a natural β-triketone herbicide had an effect on soil fungal community α- and β-diversity underlining the need to even assess the ecotoxicity of biocontrol product on non-target organisms using microbial endpoints.

## Author Contributions

All authors contributed to the design of the work or the acquisition, analysis, or interpretation of data for the work. CM, SR, CC, FM-L, and LB were involved in drafting the work and critical reading of the manuscript and final approval of the version to be published.

## Conflict of Interest Statement

The authors declare that the research was conducted in the absence of any commercial or financial relationships that could be construed as a potential conflict of interest.

## Supplementary Material

TABLE S1 OTU table.
